# Managing social-educational robotics for students with autism spectrum disorder through business model canvas and customer discovery

**DOI:** 10.3389/frobt.2024.1328467

**Published:** 2024-04-24

**Authors:** Anshu Saxena Arora, Amit Arora, K. Sivakumar, John R. McIntyre

**Affiliations:** ^1^ Department of Management, School of Business and Public Administration, University of the District of Columbia, Washington, DC, United States; ^2^ Department of Marketing, College of Business, Lehigh University, Bethlehem, PA, United States; ^3^ Center for International Business Education and Research (CIBER), Georgia Institute of Technology, Atlanta, GA, United States

**Keywords:** social-educational robots, robotic interventions, business model canvas (BMC), customer discovery, autism spectrum disorder (ASD)

## Abstract

Social-educational robotics, such as NAO humanoid robots with social, anthropomorphic, humanlike features, are tools for learning, education, and addressing developmental disorders (e.g., autism spectrum disorder or ASD) through social and collaborative robotic interactions and interventions. There are significant gaps at the intersection of social robotics and autism research dealing with how robotic technology helps ASD individuals with their social, emotional, and communication needs, and supports teachers who engage with ASD students. This research aims to (a) obtain new scientific knowledge on social-educational robotics by exploring the usage of social robots (especially humanoids) and robotic interventions with ASD students at high schools through an ASD student–teacher co-working with social robot–social robotic interactions triad framework; (b) utilize Business Model Canvas (BMC) methodology for robot design and curriculum development targeted at ASD students; and (c) connect interdisciplinary areas of consumer behavior research, social robotics, and human-robot interaction using customer discovery interviews for bridging the gap between academic research on social robotics on the one hand, and industry development and customers on the other. The customer discovery process in this research results in eight core research propositions delineating the contexts that enable a higher quality learning environment corresponding with ASD students’ learning requirements through the use of social robots and preparing them for future learning and workforce environments.

## 1 Introduction

Social-educational robotics and technological advancements in human-robot interaction (HRI) are revolutionizing education, learning, and cognitive rehabilitation capabilities (Leoste et al., 2022; Bharatharaj et al., 2023; Soleiman et al., 2023). HRI research is a rapidly expanding field of artificial intelligence (AI) encompassing science, technology, engineering, and mathematics (STEM), robotics, human-computer interaction, psychology, and social sciences. Researchers are now investigating the social, behavioral, and cognitive aspects of HRI (Newman et al., 2022).

Social robots, especially humanoids, are popular with humans due to their anthropomorphic, humanlike features and their capability to perform autonomous movements, sensory-motor tasks, and verbal and non-verbal communications (Zhang et al., 2019; Arora et al., 2022; Bertacchini et al., 2022). Social robotics researchers have defined ‘social robots’ in the HRI literature as ‘sociable’ (i.e., robots can be used as tools/aid for social cognition), ‘socially evocative’ (i.e., robots are anthropomorphic and evoke positive feelings in humans during human-robot interaction), ‘socially intelligent’ (i.e., robots portray social intelligence and exhibit models of social competence and human cognition), ‘socially situated’ (i.e., robots are intelligent beings and can distinguish objects and other social agents in their social space), and ‘socially interactive’ (i.e., robots can be utilized for peer-to-peer HRI for social interaction and interventions with humans) agents (Mahdi et al., 2022; Newman et al., 2022; Roesler, 2023).

In the Springer Handbook of Robotics, social robots are defined as being ‘human-centric’ with the capabilities of operating in human-centered environments (Breazeal et al., 2016). They can be humanoids or animal-like with a unifying feature of engaging “people in an interpersonal manner, communicating and coordinating their behavior with humans through verbal, non-verbal, and affective modalities” (Breazeal et al., 2016 p. 1936).

Autism Spectrum Disorder (ASD) is a pervasive developmental disorder characterized by abnormalities in social interaction and communication, and restricted, repetitive patterns of behavior, interests, or activities (DSM-5, 2013). Many students with ASD typically avoid direct physical contact, do not orient toward others, do not point to communicate, and do not display signs of happiness or interest (Rutter, 2011). Some individuals with ASD require a high level of assistance in their daily lives, while others may function independently. ASD usually manifests before three and can last throughout a person’s life, though symptoms may improve with age (Rutter, 2011; Rutter et al., 2011).

Our research aims to fill significant gaps in the robotics education literature in the context of HRI by focusing on how high school students diagnosed with ASD engage in instruction provided by educational-social robots. Many research studies are available for educational robotics with elementary and middle school students. Still, little research is available on high school ASD students’ motivation, cognition, and engagement with educational-social robots. Additionally, from robot design and curriculum development perspectives, there is a lack of expertise in creating a versatile methodology (e.g., Business Model Canvas or BMC) for robot design and curriculum development aimed at ASD students that can be validated through systematic investigation across educational research and industry applications. Even though many aspects of the BMC methodology, such as defining the system’s goals, participatory design, and conducting ethical research on children/adolescents are intuitive, no visual, structured, and standardized methodology like BMC is available to the HRI and ASD community. As a strategic management tool, BMC is used to develop new business models or improve existing ones. It considers key stakeholders, value propositions, infrastructure, customers, customer relationships, and finances. Such a tool can help bridge existing gaps in HRI research among robotic designers, roboticists, industry, academics, students (interacting with social-educational robots), parents, teachers, and counselors (Arora et al., 2022).

This research makes three significant contributions. First, we develop an integrative *ASD student–teacher (co-working with social robot)–social robotic interactions* triad framework that considers the social context in which robots operate with ASD students and teachers co-working with social robots and robotic technology. We offer eight core research propositions that highlight avenues for research. Robotic interactions and collaborations between humans (ASD students and teachers co-working with robots to help students with ASD) and social robots help to design and make knowledge-based, social robots and robotic technology more relevant and effective. Second, we highlight the importance and relevance of the Business Model Canvas (BMC) framework, signifying the triad of ASD student–teacher (co-working with social robot)–social robots. We conducted a series of customer discovery interviews in high school contexts with ASD students and their teachers (co-working with robots to help ASD students) in a large metropolitan area and a federal district of the United States of America to illustrate the BMC framework’s relevance. Third, we will help connect the interdisciplinary fields of consumer behavior research, AI, social robotics, and human-robot interaction (HRI). Through this research, we wish to illustrate how the field of social robotics is helping to shape a sustainable future involving neurodivergent ASD individuals, which is far beyond the mere replacement of human workers.

In this research, we propose a conceptual framework through a business model canvas methodology and customer discovery interviews of key stakeholders engaged in social robotic interactions with ASD students. Our study aims to target the 2030 Sustainable Development Goals (SDGs) of the United Nations Organization, which were developed as an internationally agreed “plan of action for people, planet and prosperity,” especially item 3–Good health and wellbeing (ensuring healthy lives and wellbeing at all ages), and item 4–Quality education (ensuring inclusive and equitable quality education and promoting lifelong learning opportunities for all). In this investigation, we broaden the research focus by considering the combinational, complex dynamics of the Business Model Canvas (BMC incorporating the triad: ASD student–teacher (co-working with social robot)–social robotic interactions framework.

The rest of the paper is organized as follows. The upcoming sections focus on the literature review followed by business model canvas methodology and customer discovery interviews addressing previously mentioned research questions. After that, we focus on our conceptual framework: ASD student–teacher (co-working with social robot)–social robotic interactions triad framework leading to the development of research propositions. Lastly, we present conclusions, limitations, and future research directions.

## 2 Social robotics and autism: a review of literature

Social robots are proven to help both typically developing (TD) students and students with autism spectrum disorder (ASD) (Chevallier et al., 2012), a neurodevelopmental disorder characterized by social communication impairments and abnormal (repetitive) behaviors (DSM-5, 2013). For example, social robots can enhance engagement and motivation, promote personalized learning, and encourage STEM education for TD students (Zhang et al., 2019; Arora et al., 2023). In contrast, for ASD students, they offer a safe and predictable environment for social interaction training, provide consistent and repetitive practice sessions, and can be customized to address individual needs, thus reducing overstimulation. These benefits, underscored by previous research (e.g., Chevallier et al., 2012; Belpaeme and Tanaka, 2021; Arora et al., 2023) highlight the versatility and effectiveness of social robots in educational settings, catering to the diverse needs of students across the spectrum of development. The development of ASD-specific social robots can be traced back to the seminal study by Emanuel and Weir (1976), in which a computer-controlled electrotechnical device, a turtle-like robot (LOGO) moving on wheels around the floor, was used as a remedial tool for a student diagnosed with ASD. It was not until the late 1990s that numerous laboratories started investigating this topic (see Begum et al., 2016; Ismail et al., 2019; Leoste et al., 2022; Bertacchini et al., 2022; Soleiman et al., 2023; Bharatharaj et al., 2023 for reviews). In the current research, a ‘student diagnosed with ASD’ is referred to as an ‘ASD student.’

As stated earlier, ASD is a pervasive developmental disorder, and it affects social interaction, communication, and behavior development, impacting each person differently and to varying degrees of severity, as the word “spectrum” implies. ASD can appear in any order and range from mild to severe. In the social context of high schools, communication and engagement challenges can lead to social isolation and bullying for ASD adolescents (Humphrey and Symes, 2010; Salhi et al., 2022). Adolescence offers an increasing self-awareness of social challenges for some students with autism, and negative encounters with peers can intensify social anxiety (White et al., 2011). Healthy peer interactions have been shown to enhance positive social/academic results (Lynch et al., 2013). Social issues are a significant obstacle to high school adolescents with ASD in achieving their scholastic goals (Camarena and Sarigiani, 2009). Since most social encounters occur outside of the classroom, in the hallways, in school cafeterias, and during extracurricular activities, the more challenging aspects of social life for students with ASD (e.g., entering social circles, making friends, and cultivating intimate relationships, *etc.*) may go unaddressed or overlooked by teachers and administrators.

Social Motivation Theory (SMT: Chevallier et al., 2012) highlights that ASD students usually prefer nonhuman and mechanical stimuli rather than seeking out or maintaining relationships with human partners (Fosch-Villaronga and Heldeweg, 2018; Tavakoli, Carriere, and Torabi, 202; Burns et al., 2022). Social interaction challenges for ASD students stem from abnormal processing of social rewards, leading to decreased attention towards social cues early on. This diminished social focus then hinders the acquisition of social skills by limiting exposure to social learning experiences, consequently contributing to difficulties in social communication and interaction (Chevallier et al., 2012; Fosch-Villaronga and Heldeweg, 2018; Tavakoli et al., 2020). SMT utilizes three socio-biological mechanisms targeting ASD students.

• Robots represent “social agents” that can move in a three-dimensional space and physically interact with people and the environment through social orienting.
• Adjustable sensory-cognitive stimulation can promote a more significant perceptive experience as a social reward than a simple video game.
• A robotic system is perceived as an “artificially intelligent humanlike agent” that can simulate human behavior in social-affective development through social maintenance, guiding ASD students in the complex world of social interactions (Chevallier et al., 2012; Burns et al., 2022).

Students with ASD show minimal activation of the brain’s reward system in response to social reinforcement, unlike their typically developing (TD) peers, for whom social interactions are inherently rewarding (Chevallier et al., 2012). To simulate social interaction between humans, humanoid (anthropomorphic) robots should integrate the social motivation mechanisms of the human brain for an effective HRI (Arora and Arora, 2020; Arora et al., 2022; Bertacchini et al., 2022; Leoste et al., 2022; Newman et al., 2022; Salhi et al., 2022; Bharatharaj et al., 2023; Soleiman et al., 2023). Given the student’s ASD characteristics, it appears worthwhile to investigate whether a social robot, with its motivational appeal, behavioral repetition, simplified appearance, and lack of social judgment, might appeal more to people with ASD than humans (Bertacchini et al., 2022; Leoste et al., 2022; Salhi et al., 2022; Bharatharaj et al., 2023; Soleiman et al., 2023).

The increased popularity/interest in robotics education has raised questions about its performance and efficiency for students (Chandra, 2014; Somyürek, 2015; Berry et al., 2016; Nemiro et al., 2017; Bertacchini et al., 2022; Leoste et al., 2022; Salhi et al., 2022; Bharatharaj et al., 2023; Soleiman et al., 2023). ASD students exhibit favorable outcomes when engaging with social robots during HRI field experiments, attributed to the social motivation theory of autism, highlighting the role of SMT in understanding the interactions between ASD individuals and robots (Dubois-Sage et al., 2024). Some HRI benefits to these students include high levels of interest, elevated attention, high engagement, calm/active behaviors (with less repetitive behavior portrayals), and emotional response modification while being comfortably engaged in an activity/instruction provided by social robots (Dautenhahn and Billard, 2002; Kozima, Nakagawa and Yasuda, 2005; Lee et al., 2012; Scassellati, Admoni and Matarić, 2012; Costescu et al., 2014). These robotic interventions and interactions result in positive learning environments for students diagnosed with ASD and other learning disorders and disabilities (Zhang et al., 2019; Arora et al., 2022; Bertacchini et al., 2022; Leoste et al., 2022; Salhi et al., 2022; Bharatharaj et al., 2023; Soleiman et al., 2023).

The social motivation theory of autism (SMT: Chevallier et al., 2012) suggests that individuals with ASD may have impaired social motivation, affecting their social learning and interactions. We can connect this theory with the Business Model Canvas (BMC) methodology by understanding the social motivation challenges faced by individuals with ASD in the business context. The Business Model Canvas (BMC) is a strategic management tool/methodology that allows one to describe, design, challenge, invent, and pivot a business model.

Our research examines the use of business model canvas and customer discovery interviews to develop responsive robotics education for high school students with ASD. One of the research questions is: how can we use customer discovery interviews and the associated inquiry processes to develop responsive robotics education through the Business Model Canvas (BMC) to capture all stakeholders in the robotic intervention process with ASD students? We’ll address this in the following sections.

## 3 Research methodology

### 3.1 Business model canvas (BMC) as a research methodology

Business Model Canvas (BMC) is a research-based, industry-oriented framework highlighting key partners, key activities, and resources related to the research, value propositions, customer relationships, customer segments, and channels (as shown in Table 1). The BMC framework integrates user experience (UX) at its core, emphasizing UX best practices to develop responsive, ethical-educational-social robots that are commercially viable in HRI situations. As the HRI literature points out, “there is a lack of expertise in integrating and adapting UX best practices and defining UX goals in the context of HRI” (Nielsen et al., 2021, p. 266). The BMC seeks to address the gaps in the literature by providing a flexible, industry-oriented framework for developing and designing ethical robots or an ethical curriculum for educational-social robots. A business model canvas is developed to design ethical robots engaged in robotic interventions for high school and university students with learning disabilities (refer to Table 1).

**TABLE 1:** Business model canvas (BMC) framework signifying the triad framework.

Key Partners/Stakeholders (triads)	Key activities	Value propositions	Customer relationships	Customer segments
Public School System–ASD High School Students, and Teachers co-working with robotic technology	Utilizing customer discovery interviews and the associated inquiry processes through the Business Model Canvas (BMC) framework to capture stakeholders’ role in robotic interventions/HRI field experiments targeted at students with ASD	Increase the engagement of students with ASD through social robotics	Letters of Support and Collaboration from High Schools in a large metropolitan area and a federal district of the United States	END-USER
• Students diagnosed with ASD @ High Schools requiring HRI				Public Schools’ System: High Schools in a large metropolitan area and a federal district of the United States
• Teachers co-working with social robots/robotic technology	• Developing Curriculum-Related Robotic Interactions/Interventions/HRI Field Experiments for Students with Autism Spectrum Disorder (ASD) in public schools	Better time management and improved efficiency for students (diagnosed with ASD) through engagement with social robotics	Memoranda of Understanding (MOUs) with Robotic Companies	PARTNERS AND INTERMEDIARIES Universities and Colleges
Robotic Companies: Robotic Companies (Supplier of NAO and Pepper Humanoid Robots used in this research)	Key Resources	Increase robotic companies’ revenue through potential partnerships with K-12 schools and universities	Channels	INFLUENCERS Parents, Associations (e.g., PTAs), and Technology Heads of Schools
	• School of Business and Public Administration			
				ECONOMIC BUYER
			Academic Researchers play a DUAL role: PARTNERS to K-12 Systems and Robotic Companies; INTERMEDI-ARIES for Robotic Companies to access public schools for selling robots/robotic technology	
	• School of Engineering and Applied Sciences			
				Robotic Companies
	Mechatronics Lab			
	• Social Robotics - Behavioral Research Lab and • Robotic Companies			

***Our Impact of this Current Research is not just on the K-12 School System but also on the Robotic Companies.

Table 1 highlights the key partners of the BMC Framework, including the Public School System (primarily middle and high schools), ASD students and teachers, and robotic companies. Our first value proposition is to increase the engagement of ASD students through social robotics. Our second value proposition is to increase robotic companies’ revenue through potential partnerships with K-12 schools. Our third value proposition is to help minimize the time for engagement of ASD students in schools and universities through our recommended technology/robotics. The potential impact would extend beyond robotics companies to the entire K-12 school system. The customer segments include public schools, ASD students and teachers, parents, associations, technology heads (as Influencers), and robotic companies (e.g., RobotLAB from San Francisco, CA) as economic buyers and partners.

*Application of Business Model Canvas (BMC) in Human-Robot Interaction (HRI) Design.* BMC methodology has been used in previous research related to robotics. Metelskaia et al. (2018) examined a specialized BMC for AI solutions in the context of robotics and AI. This framework is instrumental in aligning AI engineering, including HRI design, with broader business strategies. The study emphasized the importance of integrating technical development with market-oriented approaches, a highly applicable principle to HRI design (Metelskaia et al., 2018). This research presented BMC as a useful tool for creating and analyzing robotic and AI solutions. Exploring the dynamic aspects of BMC, Romero et al. (2015) presented an enriched BMC design using system dynamics. This approach offered a more nuanced understanding of the complexities involved in HRI design, emphasizing the flow network and the potential for identifying and testing changes in the business model. This modified approach showed additional benefits that can be obtained with its application.

Zec et al. (2014) discussed the strengths and limitations of the BMC approach in collaborative environments. Their analysis provided insights into how software support can enhance collaborative design and evaluation of business models, a concept that can be extrapolated to collaborative HRI design processes. Bätz and Siegfried (2022) critically examined BMC’s use in entrepreneurial contexts, suggesting that it might oversimplify the multifaceted nature of business environments, such as those in robotics. This critique is crucial in assessing BMC’s applicability in the intersecting domains of technology, human interaction, and business goals. Joyce and Paquin (2016) introduced a triple-layered business model canvas, adding environmental and social layers to the traditional BMC. This extension is particularly relevant for HRI design, underscoring the need for sustainable and socially responsible robotics solutions.

Despite the above shortcomings, BMC methodology offers a viable and effective framework to understand the applicability of social robotics for students with ASD. Even though the research on BMC and HRI environments is limited, the application of BMC in HRI design offers a comprehensive framework for aligning robotic technology with strategic business objectives. Previous research highlights the versatility of BMC in addressing diverse aspects of HRI design, from enhancing learning environments to ensuring sustainability and social responsibility. The convergence of BMC and HRI design has the potential to pave the way for more integrated, effective, and responsible robotic solutions in various sectors. Our research directly applies BMC methodology aided by customer discovery interviews to develop responsive robotics education for high school students with ASD.

*Business Model Canvas (BMC) and Social Motivation Theory of Autism (SMT).* When considering the connection between SMT and BMC, it is important to integrate the understanding of social motivation challenges faced by ASD individuals into the various elements of the business model. For instance, in the customer segments and customer relationships sections, businesses can consider how to adapt their approaches to account for the social motivation difficulties of ASD individuals. This may involve creating inclusive and accessible customer experiences and communication strategies.

Furthermore, in the key activities and resources sections of the BMC framework, businesses can explore how to support employees with ASD by providing appropriate accommodations that consider their social motivation challenges. This may involve tailored training programs, workspace adjustments, and communication support. By integrating the principles of the SMT into BMC, businesses can work towards creating more inclusive environments for ASD individuals, thereby tapping into a potentially underutilized talent pool, and better serving a diverse customer base. For the value propositions section of BMC, we need to develop a deeper understanding of SMT that resonates with individuals with ASD, such as creating environments or products that are less overwhelming and more accommodating to sensory sensitivities. SMT can influence the choice of channels used to reach out to ASD individuals, opting for those that are more aligned with their social preferences and comfort zones. Adapting a business model to cater to ASD individuals might involve unique cost considerations. Still, it could also open up new revenue streams by tapping into an often underserved market.

### 3.2 Customer discovery interviews

Customer discovery interviews are a crucial component of the business model canvas (BMC) research methodology, particularly in the field of social robotics and human-robot interaction (HRI) (Arora et al., 2023). These interviews involve engaging with potential customers to understand their needs, preferences, and problems (or pain points), which can then be used to inform the development of a business model. In the context of social robotics and HRI, customer discovery interviews can provide valuable insights into the specific use cases and applications of robots in various industries, such as hospitality and tourism (Tung and Au, 2018; de Kervenoael et al., 2020). For example, Tung and Au’s (2018) study on consumer experiences with robotics in hospitality highlights the influence of robotic embodiment and human-oriented perceptions on consumer experiences, which can offer valuable insights for businesses in this sector. Similarly, de Kervenoael’s et al. (2020) work on visitors’ intentions to use social robots in hospitality services underscores the importance of perceived value, empathy, and information sharing in driving these intentions, providing further guidance for businesses in this field.

The BMC methodology incorporates insights gathered from customer discovery interviews, ensuring that user needs and preferences are considered during the design and development process (Arora et al., 2023). These customer discovery insights can then be integrated into the BMC methodology to develop a sustainable and effective business model for social robotics and HRI. BMC’s visual representation (refer to Table 1) encourages collaboration among team members, providing a clear and concise representation of the social robot’s components and their interrelationships. BMC’s modular structure enables researchers and developers to easily modify and update different aspects of the social robot as new insights or technological advancements emerge (Alves-Oliveira et al., 2022; Arora et al., 2023). By utilizing the BMC, researchers can create a visual representation of the various components that help in a successful social robot interaction and implementation, including customer segments, value propositions, channels, and revenue streams (Arora et al., 2023). By combining customer discovery interviews with the BMC methodology, robotic creators, developers, and researchers can create social robots that are not only technologically advanced but also user-centric, maximizing user experience and ultimately leading to more successful and effective HRI implementation (Alves-Oliveira et al., 2022).

We conducted two studies utilizing customer discovery interviews. Study one engages ASD students and their teachers, whereby a total of 25 customer discovery interviews were conducted. On the other hand, Study two involves other stakeholders from schools (e.g., school principals, technology heads, *etc.*) in addition to the industry professionals from robotic companies, whereby a total of 35 customer discovery interviews were conducted. Study one is described below. Study two is described later, under Section 5.3.

*Study 1: Participants and Educational Settings*. We conducted sixteen customer discovery interviews with ASD students from high schools after they interacted with social robots during HRI field experiments or social robotic intervention sessions. We also conducted nine interviews with their teachers, who had interacted with both robots and students. These interviews were conducted at three different public high schools of a large metropolitan federal district in the United States. We used Individualized Educational Plans (IEPs) to recruit students in consultation with the school counselors and teachers. A student’s IEP confirmed the recruited participant had a professional diagnosis of ASD and that the teachers interviewed were aware of the students’ ASD diagnosis. The high school students were 15–17 years old, with 11 males and five females. We used two kinds of social robots: NAO and Pepper. Both robots easily create an empathetic link with students, teachers, and researchers through their eye-catching appearances, moderate sizes, and humanoid behaviors.^1^ Our research proposal, including its objectives, methodologies, and participant engagement strategies, was approved by the Institutional Review Board.

*Procedure.* Five sessions (1 hour each) were conducted using the social robots with 16 ASD high school students (see Supplementary Appendix S1). At the end of the session, researchers filled out an evaluation form with five variables (e.g., focused attention, following instructions, physical and verbal imitation, emotional response, and performance). Parents were informed of the study with their due consent taken before the study. The inclusion criteria for student selection were: (a) high school students diagnosed with ASD, (b) between 15 and 17 years of age, (c) obtained ‘informed consent’ signed by their parents, and (d) selected by high school counselors for HRI experiments with social robots according to their respective IEPs. The exclusion criteria were: (a) high school students who did not meet the age criteria (15–17 years of age), (b) did not obtain ‘informed consent’ from their parents, and (c) students with hearing, speech, and vision deficits, with abnormal eye movements and comorbidities such as Fragile X Syndrome or Down’s Syndrome, and/or students diagnosed with other learning disorders.

Supplementary Appendix S1 provides information on the demographics of ASD students and teachers, and Supplementary Appendix S2 provides more detail about the interview procedure and questions. Both sets of interviews included questions dealing with task accomplishment according to curriculum development for social-emotional skills targeting ASD students, and the interpersonal/people dimension of the task focused on social-emotional skills development. Multiple robotic intervention sessions were conducted with these 16 ASD students. The curriculum-related, educational-ethical robotic intervention scenarios focused on social-emotional learning (SEL) skills--comfort zone, conflict resolution, and job search -- were developed as a part of the current research. These newly developed curriculum-related robotic intervention scenarios include:

• **Comfort Zone:** This human-robot activity introduces humans (ASD individuals) to the concept of a comfort zone through the social robot and explains its benefit to the ASD individual.
• **Conflict Resolution:** This human-robot activity explores the skill of conflict resolution, such that it involves helping someone resolve a conflict within themselves or between others: communicate, compromise, ask for help, apologize, and write a Pro and Con list through the social robot.
• **Job Search:** This activity explores the skill of job search (explained to the human/ASD individual through the robot) and making use of available job opportunities: talking with others, business signs, community support, the internet, and/or newspapers.

Lessons on moral values and ethics were integrated for the ASD students in each SEL skill. These human-robot activities were developed as case-based, ethical curriculum-related robotic intervention scenarios and individual lesson plans for students with ASD and other learning disabilities^2^ (see Arora et al., 2022).

Interviews have been used in previous research across disciplines as a robust method for formulating research propositions. Specifically, van Doorn et al. (2023) leverage the power of qualitative interviews to explore significant relationships at the intersection of consumers, autonomous technology, and workers. This approach is pivotal in developing their Consumer–Autonomous Technology–Worker (CAW) framework, which sheds light on the evolving landscape of organizational frontlines in the digital age. By engaging directly with workers co-working with robots and consumers interacting with these human-robot teams, van Doorn et al. (2023) gather rich, contextually nuanced insights that are critical for framing their research propositions.

All interviews were transcribed by the researchers to ensure that the rich, qualitative data contained within could be analyzed. Our methodology was informed by the principles and practices suggested by van Doorn et al. (2023), tailored to explore the specific nuances and dynamics observed in HRI settings. Our analytical process involved a detailed examination of the transcriptions to identify key patterns, behaviors, and insights related to the interaction between students and social robots. This involved an iterative process of coding the data (first order codes, second order codes, and aggregate dimension) as per Gioia approach (Gioia et al., 2013), discussing emergent patterns among the research team, and refining our understanding of the data considering the broader literature and the specific objectives of our research. In Table 2, we provide an example of our coding.

**TABLE 2:** Coding example (Gioia et al., 2013; van Doorn et al., 2023).

1st order code	Quote	2nd order code	Aggregate dimension
**ASD student’s responses to social robots via teachers**	“*I wait to meet my NAO on a regular basis. Initially, there were university professors who introduced us to NAO. Now, when my teacher uses NAO and brings it with her, I feel very happy. I feel that robots are our common interests.”*	**Human-Robot Interaction** (**HRI**) **Experiments of ASD students interacting with social robots, collaborated and supported by their teachers**	**Changes in Teacher-Student Dynamics** (Role and Impact of Learning Experience) in schools when facilitated with social robots
**Teacher’s opinions of Student-Robot Interactions**	*“When my student interacts with NAO or Pepper directly, of course, s/he enjoys the interaction. However, when I bring the robot with me, I find my students happier than them enjoying the interaction without me.*”	(How do ASD students (and their teachers) experience overall learning experience with the social robots)	

In line with the approach taken by van Doorn et al. (2023), we use customer discovery interviews in our study to develop impactful research propositions. By engaging in direct conversations with students, teachers, and robotic professionals from the industry, we gain access to their lived experiences and insights, which are crucial for grounding our research propositions in real-world contexts. This approach aligns with the qualitative research tradition, where the depth and richness of data gathered through interviews offer a robust foundation for developing meaningful and relevant research propositions. Thus, the methodology employed in our study, inspired by van Doorn et al.’s (2023) work, stands on solid academic ground, demonstrating that customer discovery interviews are powerful instruments for generating deep insights essential for scholarly research.

## 4 Conceptual framework: ASD student–teacher–robot triad framework

A social robot can be designed along a spectrum of autonomy—ranging from non-autonomous or Wizard-of-Oz designed, semi-autonomous, to fully autonomous. Autonomous technology is defined as “machines capable of performing actions without (or with minimal) human intervention that can change their behavior in response to unanticipated events (Watson and Scheidt, 2005) … developed remarkably over recent decades and has become a top priority of both researchers and managers” (van Doorn et al., 2023, p. 2). Much research is available on consumer-facing AT (e.g., chatbots or digital voice assistants such as Alexa and Siri) that help consumers select the right goods and services (Guha et al., 2021). In this research, embodied robots (e.g., Pepper) guiding consumers in a store, a building, or school premises are considered consumer-facing AT. Employee (or worker)-facing AT can be considered a medical AI assisting hospital doctors (Longoni et al., 2019). In our research scenario, teachers working and collaborating with NAO and Pepper robots to teach ASD students can be considered employee-facing AT.

**FIGURE 1:**
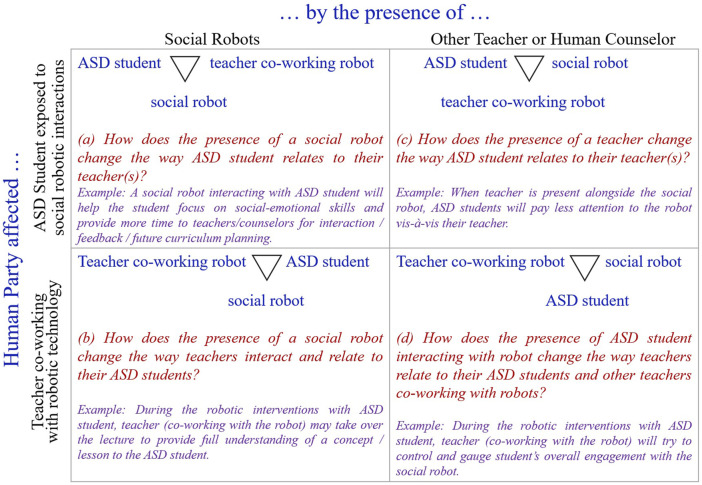
Relationships within the Triad [ASD Student–Teacher (co-working with social robot)–Social Robot] Framework (Adapted from van Doorn et al., 2023).

Utilizing van Doorn et al.’s (2023) framework for consumer-facing AT and worker-facing AT, we propose our ASD student–teacher (co-working with social robot)–social robot triad framework (refer to Figure 1) with eight core research propositions. Figure 1 illustrates the relationships between ASD students and their teachers and how these relations change when social robots are integrated into curriculum planning for ASD students. We acknowledge that reality may be more complex than these portrayed relationships between ASD students and their teachers.

In each quadrant of Figure 1, we provide anecdotal evidence from a series of interviews conducted during human-robot interaction (HRI) field experiments or social robotic intervention sessions between the robots and the ASD students. Both ASD students’ and teachers’ interviews focused on social robotic interventions (or HRI field experiments). They emphasized the need for collaboration between the teacher and their ASD student in a way that the ASD student–teacher (co-working with social robot)–social robot triad is considered as a whole.

Utilizing the Business Model Canvas (BMC) framework to signify the triad of ASD student–teacher (co-working with social robot)–social robot (as seen in Figure 1), we develop eight research propositions in the next section. We start by focusing on ASD student–social robot and teacher (co-working with social robot)–social robot dyads. Each quadrant of the ASD student–teacher (co-working with social robot)–social robot triad framework (Figure 1) is explained in detail in the following section. Thereafter, we bring the three actors together in the triad framework by offering eight core research propositions.

## 5 Development of research propositions

### 5.1 Teacher–student relationship through the social robot: how does the presence of a ‘social robot’ change (a) the way ASD students relate to their teachers, and (b) the way teachers interact and relate to ASD students?

This sub-section focuses on the top-left and bottom-left quadrants of the ASD student–teacher (co-working with social robot)–social robot triad framework (see Figure 1). Top-left quadrant focuses on the relationship between ASD student and teacher (co-working with robot) through the presence and use of a social robot. The presence of a social robot helps both ASD students and their teachers focus on social-emotional skills and provides more time for teachers and counselors to engage in interaction, feedback, and future curriculum planning. Social robots can help ASD students engage with the learning material by providing a novel and interactive learning experience (Belpaeme and Tanaka, 2021). This aspect can be particularly beneficial for students with ASD, who may struggle with traditional teaching methods, and indicates that social robots can play a supportive role in strengthening the ASD student-teacher relationship in the context of ASD education (refer to the top-left quadrant of Figure 1).

The bottom-left quadrant focuses on the relationship between teacher (co-working with robot) and ASD student through the presence and use of a social robot. When ASD students are exposed to social robotic interactions through their teachers, it may affect how they relate to their teachers and how their teachers interact and relate to the students. Teachers may frequently switch between different perspectives when interacting with social robots, such as viewing the robot as a didactic tool or a social actor (Ekström and Pareto, 2022). This flexibility could potentially help teachers adapt their teaching strategies to support ASD students better. Teachers may try to create an inclusive approach, encourage collaboration, and establish mutual trust between the actors in their assigned roles (Ekström and Pareto, 2022). This could lead to a more supportive and inclusive learning environment for ASD students created by their teachers through social robots (refer to the bottom-left quadrant of Figure 1).

Social-educational robotics is an innovative and viable platform for:

• teaching and learning science, technology, engineering, and mathematics (STEM) and STEM-related curricula across diverse disciplines;
• developing broad learning skills such as scientific inquiry, engineering design, problem-solving, creative thinking, and teamwork; and
• fostering students’ motivation to engage in science and technology while reducing psychological and cultural barriers for minority students from underprivileged communities.

Robotics education can drive students to behave as co-constructors of learning rather than passive knowledge receivers or technology consumers. Broadening involvement in STEM education is essential for providing equitable learning opportunities for students with varying needs and diverse backgrounds (Lee and Buxton, 2010; Bellas and Sousa, 2023). Social-educational robots may serve as social mediators, encouraging prosocial behaviors in interactions with individuals. These behaviors include orienting the eyes and head, initiating physical contact, and pointing to shared interests (Dautenhahn et al., 2003; Diehl et al., 2012). Thus, our central hypothesis revolves around applying the social motivation theory of ASD to social-educational robotics. Our research focuses on students diagnosed with ASD, referred to as ASD students. It aims to understand how these ASD individuals positively react to sensory rewards delivered by a social robot, indicating their interest and satisfaction exposed to these stimuli (Kostrubiec and Kruck, 2020; Bellas and Sousa, 2023).

During our interviews with ASD students, we found that students enjoyed interacting with robots more than their teachers or counselors. However, when teachers incorporated social robots into the classroom curriculum, ASD students enjoyed interacting with their teachers and the robots. One teacher interviewee articulated this by stating that “*While social robots take care of the routine task (lesson plan) accomplishment of teaching social, emotional skills (e.g., comfort zones, conflict resolution, preparing for college and job applications, etc.), teachers have extra time to focus on an individual student’s progress and growth, thus improving their relationships with their students.*” Consequently, we propose the following.

**Research Proposition 1:** Teachers are more likely to forge stronger relationships with ASD students when social robots focus on routine tasks (e.g., teaching lesson plans), and teachers focus on creative, relational tasks, leading to overall student development and growth.

While interviewing teachers and ASD students, it was evident that some teachers had concerns about robots replacing them (Bellas and Sousa, 2023). Not all teachers are comfortable working with robots; some need training to collaborate with robots and students effectively. However, when teachers overcome their fears and view robots as valuable aids rather than their replacements, they tend to relate better with their students. One teacher interviewee emphasized this by saying, “*When my student interacts with NAO or Pepper directly, of course, s/he enjoys the interaction. However, when I bring the robot with me, I find my students happier than them enjoying the interaction without me.*” Thus, we have the following research proposition.

**Research Proposition 2:** Teachers are more likely to forge weaker relationships with their ASD students when teachers (a) are not provided with adequate training to work and collaborate with robots, and (b) they fear robots replacing them in their jobs.

### 5.2 Student-Teacher Relationship through the Social Robot: How does the presence of (a) teachers collaborating with social robots affect the way ASD students relate to their teachers and robots, and (b) ASD students interacting with social robots change the way teachers relate to their ASD students and robots?

In this sub-section, we focus on the top right and bottom right quadrants of the ASD student–teacher (co-working with social robot)–social robot triad framework (see Figure 1). The top-right quadrant focuses on the relationship between an ASD student and a social robot through the presence of a teacher (co-working with robot). When a teacher is present alongside the social robot, the focus is on how the teacher engages the ASD student in various activities and learning scenarios through the social robot. Teachers can provide personalized and engaging experiences that cater to the specific needs of ASD students. They can offer a range of stimuli that help ASD students improve their social interaction, communication skills, and emotional recognition. The social robot serves as a consistent and predictable assistant to the teacher, which can be especially comforting for ASD students, who often prefer structured and routine interactions. This quadrant highlights the potential of social robots as complementary tools for facilitating learning and social interaction in ASD students (Belpaeme and Tanaka, 2021).

The bottom-right quadrant of the ASD student–teacher (co-working with social robot)–social robot triad framework (see Figure 1) explores the relationship between the teacher (co-working with robot) and the social robot when the ASD student is present. During robotic interventions with ASD students, the teacher (co-working with social robot) utilizes the robot as a teaching aid, and the subsequent collaboration aids and influences the educational process. Teachers can leverage the capabilities of social robots to enhance their teaching methods, using them as tools to demonstrate concepts or as interactive elements that add novelty and engagement to lessons. Social robots also allow teachers to observe and understand how ASD students interact with technology, providing valuable insights that can be used to tailor educational approaches (Ekström and Pareto, 2022). This quadrant underscores the collaborative potential between human educators and robotic technologies in creating a more effective and inclusive educational environment for ASD students.

Social-educational robotics has proven to be successful with ASD students. However, the potential of robotics in teaching has been debated with little regard for different types of students (Alimisis, 2013). Previous research focused on what robotics concepts and skills ASD and typically developing (TD) students can learn (e.g., Bers et al., 2014; Atmatzidou and Demetriadis, 2016) rather than how they learn. When teachers use social robots by focusing on student learning outcomes, detailed descriptions of robotic kits and curricula for instructional approaches receive much attention. Still, there is insufficient explanation for how different students interacted with the robots and participated in the activities. According to Johnson (2003), “the universality of the robotics phenomenon” (p. 16) implies that robotics education is effective for all students regardless of their unique learning styles and diverse backgrounds. It is critical to investigate how children with varying needs and abilities engage in robotics to develop and implement responsive educational programs (Alimisis, 2013; Apraiz et al., 2023) to meet their needs.

**FIGURE 2:**
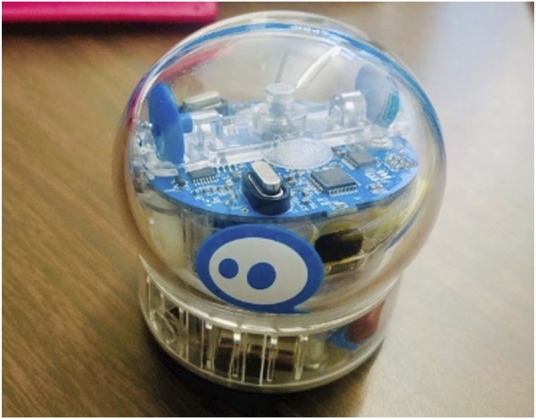
Sphero Robots (autonomous technology) in classrooms.

We interviewed the educational technology head and four of their associates for the entire public school system. We inquired about the technology assistance for students with learning disabilities. Evidently, the school system grants autonomy to individual schools to make their own technology choices. Assistive Technology is utilized through computers and/or gaming targeted at students with learning disabilities. Ensuring the safety of technology and safeguarding the privacy of students’ data is of utmost importance when engaging students with different learning disorders. In fact, the public schools’ system provides autonomous robotic technology (Sphero robots–please see Figure 2) to be used by each system school for all students. Additionally, special education teachers and counselors can request a set of 20 Sphero robots for their classrooms free of cost.

One of the ASD student interviewees (Interviewee #3) mentioned: “*I wait to meet my NAO on a regular basis. Initially, there were university professors who introduced us to NAO. Now, when my teacher uses NAO and brings it with her, I feel very happy. I feel that robots are our common interests.*” Students enjoy the overall learning experience with social robots when it is facilitated by their teachers. Thus, we have the following research proposition.

**Research Proposition 3:** ASD students are more likely to forge stronger relationships with their teachers when teachers co-work and collaborate with social robots for teaching purposes due to shared humanness, interests, and engagement.

However, in a different situation, if the teacher relies heavily on social robots without involving herself/himself in the class and engaging students, students do not enjoy the overall learning experience. One student interviewee (Interviewee #6) said, “*My teacher brings in the robot and then leaves. University researchers make us interact with the robot, but I miss my teacher’s voice. I wish she can be present when the robot interacts with us.*” Another mentioned: “*It was my first time interacting with Pepper, and my teacher left me with Pepper and other university professors/researchers. I was intimidated and immediately left the room.*” Thus, we have the following research proposition.

**Research Proposition 4:** ASD students are more likely to have weaker relationships with their teachers when student-robot interaction is (a) not mediated by their teachers, and (b) when teachers are not a part of the overall social robotic intervention process between ASD students and robots.

### 5.3 Social robots’ and robotic companies’ perspectives

Having examined the perspectives of teachers co-working with robots and ASD students interacting with social robots in the above sections, we now focus on the robotic companies’ perspectives. Critics of educational-social robotics have argued that emotional bonding created between humans and anthropomorphic robots can make people vulnerable to emotional manipulation (Zhang et al., 2019; Arora, Arora, Jentjens et al., 2022; Arora et al., 2022; Sammonds et al., 2022; Yepez et al., 2022; Apraiz et al., 2023; Roesler, 2023) and can create ethical challenges. Regulation and ethics are interrelated and are essential to regulate robotic frameworks.

Earlier standards in robotics technology separated robots from human operators for safety concerns through the European legislative via the Machine Directive 2006/42/EC, ISO 10218 (robots and robotic devices, and safety requirements for industrial robots Part 1 and 2), among other laws (Danks and London, 2017; Apraiz et al., 2023). European harmonized standards do not cover robots in educational-social spheres, autonomous vehicles, and/or additive manufacturing. Only industrial robots are a part of these standards/laws.

ISO 13482:2014 standard focuses on human-robot interaction situations of voice-controlled robotic wheelchairs, exoskeletons, and other social robots, where minimum safety requirements of HRI are defined in terms of design factors dealing with, but not limited to, robot shape, robot storage, robot motion, and other design considerations (Danks and London, 2017). Since there is a lack of standardization in incorporating safety laws and standards in robot design worldwide, there is a growing potential for developing these standards for educational-social and collaborative robotics, including service, healthcare, medical, personal care, and/or therapeutic robotics. Some of these standards deal with moral hazards associated with robots.

For example, the British Standard BS 8611:2016 on Robots and Robotic Devices enables roboticists to perform an ethical risk assessment of artificial agents. The USA-based standard, IEEE Ethically Aligned Design, mandates that roboticists and engineers should be empowered to take control of ethical design considerations in the development of robots. During the customer discovery interviews, a robotics company professional interviewee mentioned: “We have always wanted to design better ethical robots by working directly with high schools and university researchers. We want to make our robots HIPAA and FERPA compliant for use by vulnerable populations, e.g., ASD students at high schools.” Thus, we have the following research proposition.

**Research Proposition 5:** Robot-based ethical interactive intervention scenarios based on school curricula will enhance learning by ASD students.

Through the customer discovery interviews with 16 ASD students and nine teachers, we analyzed the curriculum-related, educational-ethical robotic intervention scenarios. These scenarios focused on social-emotional learning (SEL) skills, including comfort zone, conflict resolution, and job search. Pictures and videos of multiple robotic intervention sessions with some of these high school students can be found at: https://photos.app.goo.gl/9EXAW9fBfdkG5Ca5A. 13 out of 16 ASD students showed interest in interacting with robots through three lesson plans–comfort zone, conflict resolution, and job search skills. All the human-robot activities or SEL skills developed as three robotic intervention scenarios were completed in approximately 30–45-min sessions over five instances each.

ASD students with high cognitive and low social skills addressed the robot as ‘he’ or a ‘human companion.’ Conversely, ASD students with high social and low cognitive skills addressed the robot as ‘it’ or a ‘technology/tool.’ These interesting findings relate to the fact that ASD students learn by focusing on skills they lack more than the skills they possess. ASD students lacking social skills found robots to be their companions, while ASD students lacking cognitive skills found robots to be their tutors or technology tools for education. Analyzing the overall performance, we found that most ASD students understood the concepts associated with SEL skills through robotic interventions. Thus, we have the following research proposition.

**Research Proposition 6:** ASD students with high cognitive and low social skills are more likely to address the robot as a ‘human’ companion. In contrast, ASD students with low cognitive and high social skills are more likely to address the robot as ‘it’ or a ‘technology/tool.’

### 5.4 Other stakeholders’ perspectives

*Study 2: Participants and Educational Settings*. In a separate study, we conducted thirty-five (35) more customer discovery interviews with schools and robotic companies. We interviewed principals, special education counselors, technology heads, and PTAs (parent-teacher associations) at three high schools in the public school system (a total of 20 interviews). Further, we interviewed 15 robotics company professionals.

*Procedure*. Supplementary Appendix S3 includes the interview questions from 35 customer discovery interviews (20 interviews with school professionals and 15 interviews with robotics company professionals) using BMC methodology. In response to the customer discovery interviews conducted at schools, a well-known middle school in the public school system (with a high focus on education and technology) reported learning disabilities and disorders (e.g., anxiety, autism spectrum disorder or ASD, attention deficit hyperactivity disorder or ADHD, learning disabilities linked to diabetes or physical medical condition) for about 20% students (259 out of 1,524 students). At least six customer discovery interviews were conducted with different stakeholders at the school: the principal, PTA president, technology head, and two special needs counselors. ASD was identified as a critical issue, and communication and educational support (CES) services were identified and provided. Two special education programs, 14 special education counselors, three social workers (one per grade level from sixth to eighth grades), and a behavioral analyst worked closely with special needs students. The school had a good focus on technology, and Assistive Technology was already in use for ASD students. Reader Pens (which read to students through iPads) and special hi-tech chairs/furniture were in use. The school system has a policy of ‘laptops for every student,’ and the educational system was found to be technology savvy (having funds/resources to use for learning and assistive technology through federal government initiatives/programs).

A highly ranked high school was selected to conduct six customer discovery interviews with school stakeholders. Of the 1800+ students, about 200 were identified with special needs and learning disabilities (Autism Spectrum with a combination of high/low social/cognitive skills). The school was using a robotic arm for educational purposes. A science teacher organized a gaming club (supported by donations from local businesses). As a part of the gaming club activities, ASD and typically developing (TD) students built gaming computers, conducted gaming activities, worked on flight simulators, and used Xbox for gaming focused on cyber security. We conducted four customer discovery interviews with the principal, technology head, and special educational counselors at another (smaller) high school with 110 students in the Engineering program. Of those students, 20–25 students were identified with learning disabilities. The school had two special needs teachers or counselors. Assistive Technology was utilized through computers.

Through the above 20 customer discovery interviews with school administrators, teachers, parents, and technology heads, we discussed the short-term and long-term impacts of human-robot interaction on ASD students, and the potential pitfalls of over-exposure, over-engagement, and over-attachment with ASD students. We received consensus about the teachers’ role and engagement in the overall social robotic intervention process with ASD students. One of the parent interviewees stated: “*I think teachers do a fabulous job in avoiding any potential negative effects of ASD students indulging with social robots.*” In another instance, a high school principal noted: “*A teacher’s presence in the classroom ensures that technology is not seen as intrusive and there is no over-indulgence with social robots.*” Teachers were found to be effective in avoiding any potentially adverse effects on ASD students indulging in social robots. Teacher engagement and collaboration with social robots help in successful human-robot interaction (HRI) implementation over the long term. Thus, we have the following proposition.

**Research Proposition 7:** Teacher (co-working with the robot) during student-robot interaction (a) helps ASD students relate more to the social robot in the short term, and (b) decreases any potential over-attachment or over-involvement (or other potential negative consequences) with the social robot over the long term.

Technology heads at schools believe that technology helps all students, but it should be safe, secure, and ethical (with privacy considerations) when engaging students with ASD and other learning disabilities. Customer discovery interviews with 15 robotic company professionals/roboticists confirm these findings. Robotic companies were keen to work with the public school system. They were open to using academic support, especially where academic researchers can act as ‘mediators’ between schools and robotic companies for designing ethical curricula for students with learning disabilities. The robotic companies were focused on HIPAA and FERPA compliances for helping students with ASD and learning disabilities. The technology head of the school system stated: “*We need to build safeguards with robotic technology. Technology (in any form) should be safe, secure, and ethical (with privacy considerations), especially while engaging students with ASD and other learning disabilities.*” School technology heads and robotic companies were happy to integrate security and privacy considerations in the robots and robotic systems through web-enabled platforms. Thus, we have the following research proposition.

**Research Proposition 8:** Ethical technological interactions will lead to better (enhanced) learning for ASD students with learning disabilities.

Table 3 highlights our eight core research propositions. Future research must empirically test these research propositions, highlighting the ASD student–teacher (co-working with social robot)–social robotic interactions triad framework. The propositions deal with the following:

• How teachers co-working with robots can forge stronger relationships with their ASD students;
• How ASD students can forge stronger or weaker relationships with their teachers;
• How social robots can impact the social and cognitive skills of ASD students through robotic interactions and interventions; and
• How can stakeholders (other than students and teachers) impact the overall triad framework?

**TABLE 3:** Research propositions within the ASD student–teacher (co-working with social robot)–social robotic interactions triad framework.

Triad member	Main idea(s)	Illustrative evidence	Research proposition
Teacher co-working the social robot	How does the presence of a ‘social robot’ change (a) the way teachers interact and relate to ASD students and (b) the way ASD students relate to their teachers?	Teacher Interviewee #2: “*While social robots take care of the routine task (lesson plan) accomplishment of teaching social-emotional skills (e.g., comfort zones, conflict resolution, preparing for college and job applications, etc.), teachers have extra time to focus on an individual student’s progress and growth, thus improving their relationships with their students*.”	Research Proposition 1: Teachers are more likely to forge stronger relationships with ASD students when social robots focus on routine tasks (e.g., teaching lesson plans), and teachers focus on creative, relational tasks, leading to overall student development and growth
		Teacher Interviewee #7: “*When my student interacts with NAO or Pepper directly, of course, s/he enjoys the interaction. However, when I bring the robot with me, I find my students happier than him/her enjoying the interaction without me.*”	Research Proposition 2: Teachers are more likely to forge weaker relationships with their ASD students when teachers are not provided with adequate training to work (and collaborate) with robots, and they fear robots as their job replacements
		Student Interviewee #3: “*I wait to meet my NAO on a regular basis. Initially, there were university professors who introduced us to NAO. Now, when my teacher uses NAO and brings it with her, I feel very happy. I feel that robots are our common interests.*”	Research Proposition 3: ASD students are more likely to forge stronger relationships with their teachers when teachers co-work and collaborate with social robots for teaching purposes due to shared humanness, interests, and engagement
		Student Interviewee #6: “*My teacher brings in the robot and then leaves. University researchers make us interact with the robot, but I miss my teacher’s voice. I wish she can be present when the robot interacts with us.*”	Research Proposition 4: ASD students are more likely to have weaker relationships with their teachers when their teachers do not mediate student-robot interaction and when teachers are not a part of the overall social robotic intervention process between ASD students and robots
Student–Teacher Relationship through the Social Robot	How does the presence of (c) a teacher (co-working with a social robot) change the way ASD students relate to their teachers, and (d) ASD students (interacting with a social robot) change the way teachers relate to their ASD students?	Student Interviewee #8: “*It was my first time interacting with Pepper, and my teacher left me with Pepper and other university professors/researchers. I was intimidated and immediately left the room.*”	Research Proposition 5: Robot-based ethical interactive intervention scenarios based on school curricula will enhance overall learning by ASD students
Social Robots’ and Robotic Companies’ Perspectives	Robotics Companies are trying to design better robots for a successful HRI implementation by receiving feedback from teachers and university researchers. Regulation and ethics are interrelated, and it is important to regulate robotic frameworks	Robotics Company Professional Interviewee: “*We have always wanted to design better ethical robots by working directly with high schools and university researchers. We want to make our robots HIPAA and FERPA compliant for use by the vulnerable populations, e.g., ASD students at high schools.*”	Research Proposition 6: ASD students with high cognitive and low social skills are more likely to address the robot as a ‘human’ companion. In contrast, ASD students with high social and low cognitive skills are more likely to address the robot as ‘it’ or a ‘technology/tool.’
Other Stakeholders’ Perspectives	Besides teachers, students, and robotic companies, customer discovery interviews were conducted for principals, special education counselors, technology heads, and school PTAs (parent-teacher associations) to understand diverse stakeholders’ perspectives	Teacher Interviewee #9: “*ASD students have varied cognitive and social skills, and that results in the way they interact with robots.*”	Research Proposition 7: A teacher (co-working with the robot) during student-robot interaction helps ASD students relate more to the social robot in the short term and avoids any potential over-attachment or over-involvement (or other potentially adverse consequences) with the social robot over the long term
		Parent Interviewee #3: “*I think teachers do a fabulous job in avoiding any potential negative effects of ASD students indulging with social robots.*”	Research Proposition 8: Ethical, technological interactions will lead to better (enhanced) learning for ASD students with learning disabilities
		Principal Interviewee # 1: “*A teacher’s presence in the classroom ensures that technology is not seen as intrusive and there is no over-indulgence with social robots.*”	
		Technology Head Interviewee # 2: “*We need to build safeguards with robotic technology. Technology (in any form) should be safe, secure, and ethical (with privacy considerations), especially while engaging students with ASD and other learning disabilities*.”	

## 6 Discussion

### 6.1 Implications of research

The human factor plays a significant role in a successful ASD student-social robot interaction mediated by a teacher’s presence. This research draws a parallel between ASD student-social robot interaction and van Doorn et al.’s (2023) research highlighting autonomous technology interaction with frontline workers and consumers, examining consumer-AT and worker-AT dyads. Our current research explored the ASD student–teacher–social robot interactions triad framework by considering the social context in which robots operate with ASD students and teachers co-collaborating with social robots and robotic technology. Building on previous literature and customer discovery interviews derived from the business model canvas (BMC) and social motivation theory of autism, we provided eight core research propositions highlighting avenues for research in the triad framework. Robotic interactions and collaborations between humans (ASD students and teachers co-working with robots to help students with ASD) and social robots help in the education (service) sector by bridging the fields of education, artificial intelligence (AI), human-robot interaction (HRI), and consumer behavior. The complex interactions between humans (ASD students, teachers) and social robots need to be studied simultaneously to understand the utilization of social robotics in the education sector. Some industry examples that could potentially work with teachers and ASD students, based on the ASD student–teacher–social robot interactions triad framework are as follows:

• Education Technology (EdTech) Companies: These companies develop and provide tools and platforms for educational purposes, including those that can be adapted for students with ASD. They may collaborate with teachers to create tailored solutions that address the unique needs of ASD students. Examples of such companies include Coursera, Blackboard, and 2U.
• Robotics Companies: Companies that specialize in developing social robots can work with teachers and ASD students to create robotic interactions that help students focus on social-emotional skills and provide more time for teachers to plan and provide feedback. Examples include iRobot, Boston Dynamics, and SoftBank Robotics.
• Special Education Service Providers: These organizations offer specialized services and support for students with ASD. They may collaborate with teachers to integrate social robots into their educational programs, helping students improve their social skills and engagement. Some examples are the May Institute, the Center for Autism and Related Disorders (CARD), and the Autism Society of America.
• Research Institutions: Universities and research centers may conduct studies and develop new technologies to enhance the education of students with ASD. They can collaborate with teachers to understand the impact of social robots on ASD students’ learning experiences and develop effective interventions.

We highlighted the relevance of the Business Model Canvas (BMC) framework, signifying the triad: ASD student–teacher (co-working with social robot)–social robots. We also conducted a series of customer discovery interviews in high schools with ASD students along with their teachers/counselors (co-working with robots to help ASD students), parents, technology heads, and robotic company professionals. Through this research, we illustrate how the field of social robotics is helping to shape a sustainable future involving neurodivergent ASD individuals, which is far beyond the mere replacement of human workers. While robotic anthropomorphism has been studied extensively, we predicted that its negative impact of over-involvement can be reduced by the presence of a human (i.e., teacher during HRI).

Through the interdisciplinary fields of consumer behavior research, AI, social robotics, and human-robot interaction (HRI), we illustrated the relevance of social robotics and how it changes the relationships between the various actors depending on a series of factors. Division of labor between social robot and teacher ensures a successful HRI for ASD students whereby technology (i.e., robot) augments HRI instead of replacing the teacher (Tsai et al., 2022; Engwall et al., 2023; van Doorn et al., 2023). Human leadership and human factors through the presence of a teacher who is comfortable collaborating with the robot help strengthen the HRI impact in the short term for the ASD student and avoid the potential pitfalls of over-exposure and over-attachment of robots with ASD students. This aligns with the social presence theory (He et al., 2012).

**FIGURE 3:**
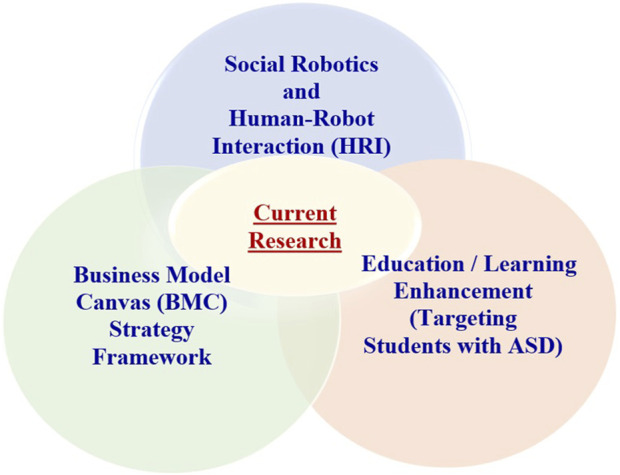
Current research: Intersection of social robotics and HRI, BMC, and learning and education.

Our research is the first to integrate the research domains of social robotics and human-robot interaction (HRI), the BMC framework, and learning and education (as depicted in Figure 3). Through the current research, we aimed to: (a) develop responsive robotics education through the Business Model Canvas (BMC) to engage all stakeholders in the robotic interventions process with ASD students, (b) create the ASD student–teacher–social robot interactions triad framework by conducting HRI field experiments with ASD students in public schools, employing the BMC, and customer discovery process, and (c) investigate how educational-social robotic interventions, specifically involving humanoid robots, contribute to the progress of high school students diagnosed with ASD and other learning/cognitive disabilities. The research involved the active participation of various stakeholders such as ASD students, teachers collaborating with robots, parents, school technology heads, and robotics company professionals.

### 6.2 Future research directions

As previously mentioned, our research aims to address two of the 2030 Sustainable Development Goals (SDGs): SDG three focuses on good health and wellbeing (ensuring healthy lives and wellbeing at all ages), and SDG 4 centers on ensuring inclusive and equitable quality education as well as promoting lifelong learning opportunities for all. There is a significant lack of awareness and understanding regarding the SDGs within the robotics community and among decision-makers. This knowledge gap is an obstacle to leveraging the contributions of robotics and AI towards achieving the SDGs (Mai et al., 2022). To overcome this challenge, future researchers should prioritize the integration of the SDGs with robotics. Additionally, there should be a stronger emphasis on interdisciplinary, human-centered, systemic thinking to highlight the benefits and relevance of social robots and robotic interventions in the context of the SDGs (Mai et al., 2022).

We acknowledge the modest sample size utilized in our study given the constraints of the novel nature of our research question, the absence of prior research in this domain, and the contextualization of our in-depth customer discovery interviews to a specific field of social robotics and HRI. Despite addressing a timely and relevant issue related to HRI, due to the modest sample size, we note that our findings must be considered as preliminary and not extrapolated beyond our research setting. However, our findings do provide some valuable insights that advance both our knowledge and practice. Furthermore, our results serve as a strong foundation for subsequent research employing larger sample sizes and examining diverse application scenarios. Future research efforts can further validate our findings and delineate boundary conditions governing our findings.

Preparing ASD students for the future is a challenging endeavor. Schools and universities are working with ASD students; however, the current effort is insufficient (Engwall et al., 2023). To address this gap, there is a growing need for more technological support (through robotics) to facilitate the development of SEL and other essential life skills like critical thinking, problem-solving, decision-making, and creative solutions. Integrating these SEL and life skills into our current educational landscape is a complex undertaking. Such integration may be achieved through HRI field experiments and building curriculum-related robotic intervention scenarios focused on life skills needed to excel in the future.

Furthermore, it is unclear whether social robots forge stronger or weaker ASD student-teacher relationships. Jackson et al. (2020) predicted stronger relationships between humans and robots where interhuman differences based on race and religion are not relevant. On the other hand, some studies show weakened interhuman relationships because humans (i.e., teachers collaborating with robots) can be potentially dehumanized (Herak et al., 2020). Future researchers should investigate the ASD student–teacher–social robot interactions triad framework provided in our study and its implications for the relationships involved.

One of the major limitations of our study is that in our ASD student–teacher–social robot interactions triad framework. We primarily examined interactions between ASD students and social robots, as well as between teachers and social robots, in individual settings. We did not explore group dynamics of decision-making within these interactions. Future researchers should investigate a broader range of research contexts.

In our research, we utilized social robots deployed by the school system (i.e., business context). However, it is important to acknowledge that in different settings, such as when robots are deployed directly by families of ASD students (a consumer context), the outcome may be different. Further, our research focused on external stakeholders. Future researchers can concentrate on robotic companies and their influence on the education sector to further advance the research enterprise. Similarly, future research should delineate the usage of social robots for neurodivergent and neurotypical employees in organizations and how social robots can impact human capital and corporate culture (van Doorn et al., 2023). Furthermore, we did not investigate our framework for its relevance to robotic companies’ suppliers, competitors, and policymakers. Future research may explore complex configurations of our ASD student–teacher–social robot interactions triad framework in diverse research contexts for different stakeholders.

We hope that our research propositions hold promise for advancing research and practice in social robotics and HRI domain, and using robotic technology to address learning disabilities in the digital age. Such progress is both timely and relevant to create a positive impact on society.

## Data Availability

The raw data supporting the conclusions of this article will be made available by the authors, without undue reservation.

## Footnotes

1
https://www.aldebaran.com/en/pepper-and-nao-robots-education

2
https://www.udc.edu/sbpa/lit/social-robotics-research/
