# Supplementary material for: Managing social-educational robotics for students with autism spectrum disorder through business model canvas and customer discovery
Source: Front Robot AI. 2024 Apr 24;11:1328467. doi: 10.3389/frobt.2024.1328467 (PMC11078017; doi:10.3389/frobt.2024.1328467)
Supplement: Supplementary file 1 [file DataSheet1.PDF]

Supplementary Material

Appendix 1

Study 1 - 25 Customer Discovery Interviews: 16 with ASD students from high schools after they interacted with social robots during HRI field experiments or social robotic intervention sessions, and 9 interviews with teachers interacting with robots and students. The high school students were in the age group of 15 – 17, 11 were males, and 5 were female students. We used two kinds of social robots: NAO and Pepper.

Overview of 25 Customer Discovery Interviews: 16 ASD Students and 9 Teachers

Interview Code	Interviewee role	School Code	Type of Robot	Experience with robots (in years)
**STU01**	ASD Student	**SCH01**	Nao	More than 1 year
**STU02**	ASD Student	**SCH03**	Nao	6 months
**STU03**	ASD Student	**SCH03**	Nao	More than 2 years
**STU04**	ASD Student	**SCH03**	Pepper	6 months
**STU05**	ASD Student	**SCH02**	Nao	6 months
**STU06**	ASD Student	**SCH03**	Nao	1 year
**STU07**	ASD Student	**SCH01**	Nao	1 year
**STU08**	ASD Student	**SCH01**	Nao	Less than 1 year
**STU09**	ASD Student	**SCH01**	Nao & Pepper	1 year
**STU10**	ASD Student	**SCH02**	Nao	More than 2 years
**STU11**	ASD Student	**SCH03**	Nao	1 year
**STU12**	ASD Student	**SCH02**	Nao	1 year
**STU13**	ASD Student	**SCH02**	Pepper	Less than 1 year
**STU14**	ASD Student	**SCH01**	Nao	Less than 6 months
**STU15**	ASD Student	**SCH02**	Nao	1 year
**STU16**	ASD Student	**SCH01**	Nao	1 year
**T01**	Teacher	**SCH02**	Nao	1 year
**T02**	Teacher	**SCH03**	Nao	1 year
**T03**	Teacher	**SCH02**	Nao	1 year
**T04**	Teacher	**SCH03**	Nao	1 year
**T05**	Teacher	**SCH02**	Nao	1 year
**T06**	Teacher	**SCH01**	Nao & Pepper	1 year
**T07**	Teacher	**SCH01**	Nao	1 year
**T08**	Teacher	**SCH03**	Nao	1 year
**T09**	Teacher	**SCH01**	Nao	1 year

Appendix 2

Interview Protocol

Given the exploratory nature of our research and the absence of prior research to answer our research questions, we utilized semi-structured (interview) questions. The goal was to keep focus on the themes relevant for our research, and not to constrain the interviewees by incorporating too much structure.

ASD Students

1. Gender / age
2. Do you need help with academics?
3. Do you need help with social skills?
4. How often and for how long do you work with social robots? How many times in a week or in a month?
5. How many times do you work with social robots along with your teachers? Are there instances when you are with robots without your teachers?
6. Do you enjoy interacting with social robots along with your teachers? Or do you prefer to interact with robots without your teachers?
7. Do you enjoy lesson plans delivered by social robots? Or only by your teachers with no robots around?
8. How does the interaction with robot affect Overall Satisfaction? Efficiency? Convenience?
9. Do you feel robot distracts you from learning rather than help you?
10. How does the robot change the way you interact with your teachers?
11. How does the robot change the way you think and perceive your school and its teachers?
12. What is the best / worst thing about having robots involved in the lesson plans? When robots are not present, what is the best / worst thing about your interactions with your teachers?
13. Is there anything you would like to mention about teachers co-working with robots and how both robots and teachers together changed your perception about learning / school / robotic interaction?
14. Any comments about: (a) social robots delivering curriculum and lesson plans, (b) teachers co-working with robots, (c) teachers not using robots at all, and (d) overall interaction experience with social robots?
15. Any more comments?

Teachers

1. How long have you worked with social robot(s)?
2. Since when did you start utilizing social robots in education? What kind of robot do you use? Describe the robot and its purpose.
3. How efficient is your school in helping you get trained with the social robot?
4. Do you utilize social robot for your curriculum or lesson plans, especially teaching social emotional skills to ASD students?
5. Do you utilize social robot for any other reason with your students apart from teaching?
6. How would you describe the collaboration between you and the robot?
7. How does working with robot affect your tasks?
8. What tasks are performed and executed by the robot that you previously conducted?

9. What additional new tasks are you executing now after you started implementing the others by social robot?
10. How does the robot impact your typical workday compared to the previous situation without a robot?
11. Do you enjoy conducting social robotic interactions and interventions for your ASD students?
12. What additional skills did you learn to collaborate with the social robot?
13. How has the robot changed the way you interact with your students? And how does the robot change the way your students interact with you or the way they perceive you?
14. Any comments about: (a) social robots delivering curriculum and lesson plans, (b) ASD students and teachers working with robots, (c) teachers not using robots at all, and (d) overall interaction experience with social robots (through the eyes of teachers)?
15. Any more comments?

Interview Procedure

Our approach aligns with the methodology outlined by van Doorn et al. (2023) in the context of the Consumer–Autonomous Technology–Worker (CAW) framework, albeit with specific adaptations to suit our research focus on Human-Robot Interaction (HRI). All interviews were transcribed by the researchers to ensure that the rich, qualitative data contained within could be analyzed. Our methodology was informed by the principles and practices suggested by van Doorn et al. (2023), tailored to explore the specific nuances and dynamics observed in HRI settings. Our analytical process involved a detailed examination of the transcriptions to identify key patterns, behaviors, and insights related to the interaction between students and social robots. This involved an iterative process of coding the data (first order codes, second order codes, and aggregate dimension) as per Gioia approach (Gioia et al., 2012), discussing emergent patterns among the research team, and refining our understanding of the data considering the broader literature and the specific objectives of our research.

Appendix 3

Study 2 - 35 Customer Discovery Interviews: 20 customer discovery interviews at three high schools within the public schools' system of a large metropolitan area and a federal district of the United States were conducted with the principals, special education counselors, technology heads, and PTAs (parent-teacher associations) of schools. Another 15 customer discovery interviews with robotics company professionals were conducted.

Overview of 35 Customer Discovery Interviews: 20 High Schools' Stakeholders and 15 Robotic Company Professionals

Interview Code	Interviewee role	School Code	Type of Robot
**STK01**	Technology Head	**SCH01**	Nao
**STK02**	Principal	**SCH03**	Nao
**STK03**	Parent	**SCH03**	Nao
**STK04**	Parent	**SCH03**	Nao
**STK05**	Parent	**SCH02**	Nao
**STK06**	Principal	**SCH03**	Nao
**STK07**	Parent	**SCH01**	Nao
**STK08**	Special Education Counselor	**SCH01**	Nao
**STK09**	Parent	**SCH01**	Nao
**STK10**	Parent	**SCH02**	Nao
**STK11**	Special Education Counselor	**SCH03**	Nao
**STK12**	Parent	**SCH02**	Nao
**STK13**	Parent	**SCH02**	Pepper
**STK14**	Special Education Counselor	**SCH01**	Nao
**STK15**	Parent	**SCH02**	Nao
**STK16**	Parent	**SCH01**	Nao
**STK17**	Technology Head	**SCH02**	Nao
**STK18**	Principal	**SCH03**	Nao
**STK19**	Parent	**SCH02**	Nao
**STK20**	Parent	**SCH03**	Nao
**COM01**	Manager	**SCH02**	Nao
**COM02**	Roboticist	**SCH01**	Nao & Pepper
**COM03**	Robot operator	**SCH01**	Nao
**COM04**	Robot operator	**SCH03**	Nao
**COM05**	Robot operator	**SCH01**	Nao
**COM06**	Robot operator	**SCH03**	Nao & Pepper
**COM07**	Manager	**SCH02**	Nao
**COM08**	Roboticist	**SCH01**	Nao
**COM09**	Robot operator	**SCH01**	Nao
**COM10**	Robot operator	**SCH03**	Nao & Pepper
**COM11**	Manager	**SCH01**	Nao
**COM12**	Roboticist	**SCH03**	Nao
**COM13**	Robot operator	**SCH02**	Nao
**COM14**	Robot operator	**SCH01**	Nao & Pepper
**COM15**	Manager	**SCH01**	Nao

<u>Interview Protocol</u>

School System – Principals, Special Needs / Special Education Counselors, and Technology Heads

1. How many students (numbers and percentages) are identified with ASD and other learning disabilities/disorders?
2. How many teachers (special education counselors and/or special needs teachers) work only with students identified with ASD and other learning disabilities?
3. What teaching aids do you use for students diagnosed with learning disabilities?
4. Do you prefer to use robots for ASD students?
5. Is there any technology in use already? – What and why?
6. How do teachers/administrative staff make decisions on technology? Any budget allocations/constraints?
7. Are parents or PTAs involved in the decision-making/technology buying process?
8. Are you happy with the technology (social robots) in place for ASD students?
9. How do you integrate robots and robotic technology at schools targeting ASD students?
10. Does the use of technology facilitate/impede the learning process?

Robotic Company Professionals

1. In your opinion, what is the best / worst thing about having robots involved in the lesson / curriculum plans?
2. How does the robot change the way teachers interact with your students? And how does the robot change the way ASD students interact with teachers or the way they perceive their own school?
3. How does the robot change the way students interact with their teachers? And how does the robot change the way students think and perceive their school and teachers?
4. Any comments about: (a) social robots delivering curriculum and lesson plans, (b) ASD students and teachers working with robots, (c) teachers not using robots at all, and (d) overall interaction experience with social robots for both students and teachers?
5. Any more comments?

Interview Procedure

Our approach aligns with the methodology outlined by van Doorn et al. (2023) in the context of the Consumer–Autonomous Technology–Worker (CAW) framework, albeit with specific adaptations to suit our research focus on Human-Robot Interaction (HRI). All interviews were transcribed by the researchers to ensure that the rich, qualitative data contained within could be analyzed. Our methodology was informed by the principles and practices suggested by van Doorn et al. (2023), tailored to explore the specific nuances and dynamics observed in HRI settings. Our analytical process involved a detailed examination of the transcriptions to identify key patterns, behaviors, and insights related to the interaction between students and social robots. This involved an iterative process of coding the data (first order codes, second order codes, and aggregate dimension) as per Gioia approach (Gioia et al., 2012), discussing emergent patterns among the research team, and refining our understanding of the data considering the broader literature and the specific objectives of our research.
